# Facile and Green Synthesis of Starfruit-Like ZIF-L, and Its Optimization Study

**DOI:** 10.3390/molecules26154416

**Published:** 2021-07-21

**Authors:** Christian J. Wijaya, Suryadi Ismadji, Hakun W. Aparamarta, Setiyo Gunawan

**Affiliations:** 1 Department of Chemical Engineering, Faculty of Industrial Technology and Systems Engineering, Institut Teknologi Sepuluh Nopember, Keputih Sukolilo, Surabaya 60111, Indonesia; ch.julius7@gmail.com (C.J.W.); hakun2397@gmail.com (H.W.A.); 2 Department of Chemical Engineering, Widya Mandala Surabaya Catholic University, Kalijudan 37, Surabaya 60114, Indonesia; suryadiismadji@yahoo.com; 3 Department of Chemical Engineering, National Taiwan University of Science and Technology, 43 Keelung Road, Sec 4, Taipei 10607, Taiwan

**Keywords:** crystal violet adsorption, healthy, optimization, starfruit-like shape, zeolitic imidazolate framework-L

## Abstract

Due to its excellent characteristics, zeolitic imidazole framework-L (ZIF-L) is widely used in various applications, such as drug delivery, wastewater treatments and energy storage. In the synthesis of ZIF-L, the molar ratio of ligand to metal, the reaction time and the temperature are essential parameters to produce excellent ZIF-L. In this work, ZIF-L was synthesized using a facile and green synthesis method. It was statistically investigated and optimized to obtain the best operating conditions. The optimization was carried out toward the amount of adsorbed crystal violet (CV) dye (q) as the response in the statistics. The optimal ZIF-L was obtained using a molar ratio of ligand to metal of 8.2220 for 97 min at 29 °C, where the q value of the CV adsorption onto this optimal ZIF-L reached 823.02 mg/g. The obtained ZIF-L was characterized using SEM, XRD, FTIR and TGA analyses to ensure its excellent characteristics.

## 1. Introduction

Metal–organic frameworks (MOFs) are advanced porous inorganic–organic materials assembled from metal clusters and organic ligands through coordination bonds [1,2,3]. They have a crystalline structure, large surface area, high pore volume, tunable pore size, good adsorption capacity, good thermal and chemical stability, and flexibility, and are easy to functionalize and modify [4,5,6,7,8]. Due to their outstanding characteristics, they have been applied in many applications, such as selective adsorption [9,10,11], water and wastewater treatments [12,13,14], composite constituents [15,16], catalysts and photocatalysts [17,18], fluorescence sensors [1,19], gas storage and separation [20,21], and drug delivery [22,23]. In the last few decades, many types of MOFs have been studied and developed through various synthesis methods, including diffusion, solvo(hydro)thermal, microwave, electrochemical, mechanochemical and sonochemical methods [24].

Zeolitic imidazolate framework-8 (ZIF-8) is a kind of ZIF that is assembled by coordinating zinc (Zn) as the metal-core and 2-methylimidazole (Hmim) as the organic ligand in the 3D structure. Previous studies have been carried out to synthesise ZIFs, especially ZIF-8, in which various surface areas, pore volumes and pore sizes were obtained [1,25,26]. ZIF-8 is called a zeolitic MOF due to its similarity to the tetrahedral framework of zeolites [15]. This allows the ZIF-8 to have a rhombic dodecahedron crystal shape [1,3,25,27]. However, it did not rule out the formation of other crystal shapes, such as hexagonal [15,28], truncated cubic [26], spherical [29], rod-like, and cylindrical crystal shapes [20]. This phenomenon may occur due to differences in the method, parameters and modulator addition. Even though ZIF-8 has excellent characteristics, the 3D structure of ZIF-8 provides a lower adsorption capacity than its 2D structure [30]. Therefore, another polymorph of ZIF-8 which has a 2D structure was developed to overcome this issue: it is called ZIF-L, and it has the same building units as common ZIF-8 [31,32,33]. Both ZIF-8 and ZIF-L have been well synthesized in aqueous systems, which are more environmentally friendly. ZIF-L needs a lower molar ratio of ligand to metal compared to ZIF-8, such that the synthesis of ZIF-L is more economical [34]. In the synthesis of ZIF-L, the molar ratio of ligand to metal plays an important role to produce ZIF-L with excellent characteristics [33,34]. In this study, two other parameters were also involved besides the molar ratio of ligand to metal in order to further optimize the synthesis of ZIF-L, namely the reaction time and temperatures. Therefore, it is important to carry out statistical process optimization. However, this statistical study has never been performed before in the synthesis of ZIF-8.

Herein, a facile, green and optimal synthesis of ZIF-L was carried out in the aqueous system. This synthesis process only used water as the solvent, which was easier to remove and safer for further applications. The molar ratio of ligand to metal, reaction time and temperature were varied in order to obtain the optimum operating conditions of ZIF-L synthesis. The effects of the parameters used were studied using the one-factor-at-a-time (OFAT) method, while the optimization study was statistically modelled using response surface methodology (RSM). Furthermore, the amount of crystal violet (CV) dye adsorbed onto the synthesized ZIF-L (q) was used as the response in the statistics.

## 2. Results and Discussion

### 2.1. One-Factor-at-a-Time (OFAT) Experiments

#### 2.1.1. Effects of the Molar Ratio of Ligand to Metal

**Figure 1:**
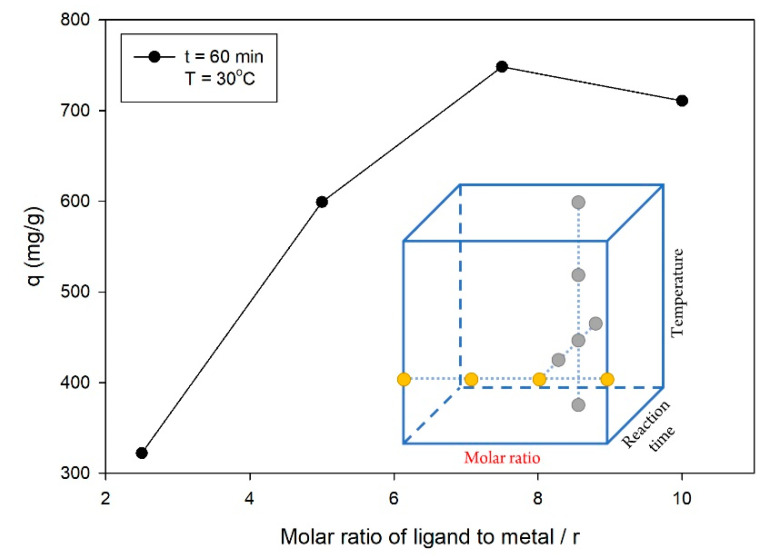
Effect of the molar ratio of ligand to the metal on the q value, with the following experimental conditions: reaction time = 60 min and temperature = 30 °C.

In this study, the synthesis of ZIF-L was focused on a lower molar ratio of ligand to metal in order to obtain a more economical synthesis process compared to the synthesis of ZIF-8. However, the reaction time and temperature were also used as other independent parameters here, in addition to the molar ratio, for the adjustment of the optimal synthesis of ZIF-L. In the OFAT experiments for the molar ratio parameter, the synthesis of ZIF-L was detained for a reaction time of 60 min at a temperature of 30 °C. As shown in Figure 1, the q value increased along with the molar ratio of ligand to metal, but it reversed down after a certain point of the molar ratio of ligand to metal. ZIF-L synthesized using a molar ratio of 7.5 had the highest q value compared to the lower and higher molar ratio. A previous study also reported that the synthesis of ZIF-8 at a lower molar ratio produced ZIF-L with a 2D structure, with a lower surface area and pore volume [35]. Therefore, the low q values of the ZIF-L synthesized here at a lower molar ratio could be hypothesized to be due to the lower surface area and pore volume. Aside from that, the use of a higher molar ratio tended to present an excess ligand initiating high initial nucleation. High numbers of nuclei were formed in the early phase due to the excess amounts of ligand, producing ZIF-L with a smaller crystal size [36]. Due to the higher q value, the molar ratio of 7.5 was used for the further steps of the OFAT experiments. However, the molar ratio of 7.5 did not exactly give the highest q value, as shown by the curve trend in Figure 1. Furthermore, the optimization was carried out using the molar ratios of 5, 7.5, and 10 in order to obtain the optimum synthesis conditions.

#### 2.1.2. Effects of Reaction Time

**Figure 2:**
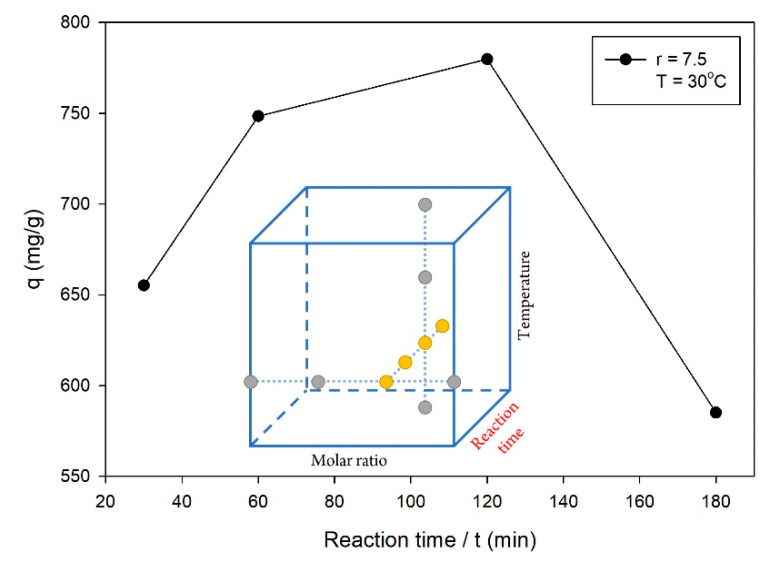
Effect of the reaction time on the q value, with the following experimental conditions: molar ratio of ligand to metal = 7.5 and temperature = 30 °C.

In the second series of OFAT experiments for the reaction time parameter, the synthesis of ZIF-L was attained using a molar ratio of 7.5 at a temperature of 30 °C. Figure 2 shows the q values for various reaction times, where the highest q value was obtained for a reaction time of 120 min among the other levels. The reaction times of 30 and 60 min were still in the crystal growth phase, such that the formation of ZIF-L had not been completed. Previous studies reported that the ZIF’s characteristics in terms of surface area and pore volume increased during the crystal growth phase until a certain time, and then remained constant [2,20]. However, Figure 2 shows the decreased q value of ZIF-L synthesized for a reaction time of 180 min. This phenomenon was suspected to occur because of the stuck residual reactants in the ZIF-L pores, which were difficult to wash off, such that the adsorption ability towards CV decreased significantly. The excess reaction time used in the synthesis allowed the adsorption of residual reactants onto ZIF-L. From these OFAT experiments, the reaction time of 120 min was used for the next step of the OFAT experiments, while the reaction times of 60, 120 and 180 min were investigated more in the optimization study.

#### 2.1.3. Effects of the Temperature

**Figure 3:**
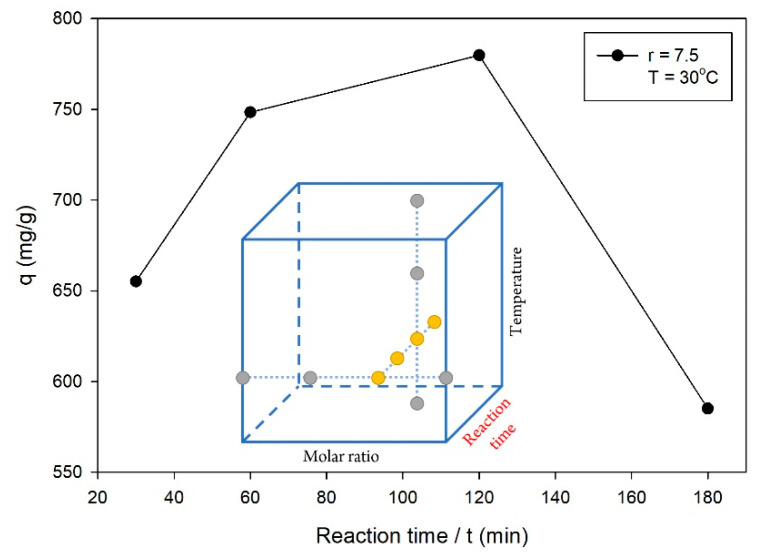
Effect of temperature on the q value, with the following experimental conditions: molar ratio of ligand to metal = 7.5 and reaction time = 120 min.

In the third series of OFAT experiments, the synthesis of ZIF-L was attained using a molar ratio of 7.5 for a reaction time of 120 min, which was obtained from the two previous series of OFAT experiments. Here, the synthesis of ZIF-L was carried out with variations in temperature, and the results are shown in Figure 3. The highest q value was obtained at a temperature of 30 °C. A previous study reported that ZIFs with a high crystallinity and surface area could be obtained at a specific temperature [20]. The crystallinity and surface area were the main characteristics that affected the q value. In the synthesis of ZIF-L, the use of a low temperature might not be able to remove the amorphous compounds in the ZIF-L, while the high temperature inhibited the formation of ZIF-L, which was shown by the lower yield of ZIF-L. The results of these OFAT experiments were used to determine the parameters in the further optimization study using RSM.

### 2.2. Response Surface Analysis

#### 2.2.1. Central Composite Design (CCD) and Analysis of Variance (ANOVA)

In this study, CCD was employed due to its ability to provide a good prediction for the entire design space in terms of the effects of linear, quadratic, and two-way interactions. The synthesis of ZIF-L with three independent parameters (the molar ratio of ligand to metal, reaction time and temperature) was designed using five levels for each parameter, as presented in Table 1. Here, the q value was still used as the response in this study. The CCD analysis presented the polynomial equation in order to express the correlation between the independent parameters and the response. The polynomial equation is presented below:

(1) $$q=798.02+52.31\left(r\right)-86.45\left(t\right)-6.82\left(T\right)-95.79\left(r^{2}\right)-112.89\left(t^{2}\right)-113.69\left(T^{2}\right)-9.05\left(r\times t\right)+14.80\left(t\times T\right)$$

where q is the amount of adsorbed CV onto ZIF-L, and r, t and T are the coded independent parameters. This equation was also used to calculate the predicted q values for each combined parameter, as presented in Table 1. A CCD analysis was successfully carried out as a statistical model to predict the correlations between the independent parameters and the response. It was evidenced by the high values of $R^{2}$, $R^{2}\left(adj\right)$ and $R^{2}\left(pred\right)$ of 99.51%, 99.40% and 99.20%, respectively.

**Figure 4:**
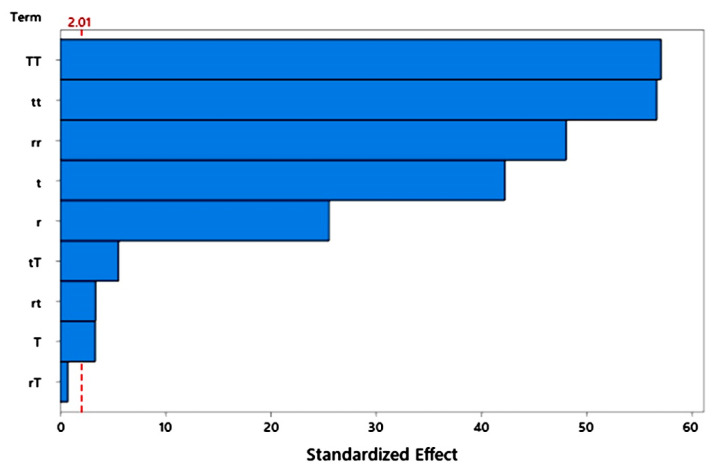
Pareto chart of the standardized effects of the independent parameters on the synthesis of ZIF-L, as measured by the q response.

Furthermore, an ANOVA study was conducted to identify the significance of three independent parameters for the response. The *F*-values and *p*-values are presented to indicate the significances in Table 2. Here, the *p*-value of the model term was significant (*p*-value < 0.05), where this means that the model used fitted the experimental data well. The significances of three independent parameters (linear) and their quadratic and two-way interactions are indicated by an *F*-value higher than its minimum limit and a *p*-value lower than 5% (0.05). As shown in Table 2, only the two-way interaction between the molar ratio and temperature (r×T) was not significant, based on a low *F*-value and a *p*-value higher than 0.05. This interpretation is also expressed in the Pareto chart (Figure 4), in which only the two-way interaction between the molar ratio and the temperature (AC) does not cross the reference line of statistical significance. Moreover, this model’s lack-of-fit was not significant due to its *p*-value higher than 0.05, as shown in Table 2, meaning that this model could precisely interpret the fit of the model on the experimental data.

#### 2.2.2. Response Surface Plots

**Figure 5:**
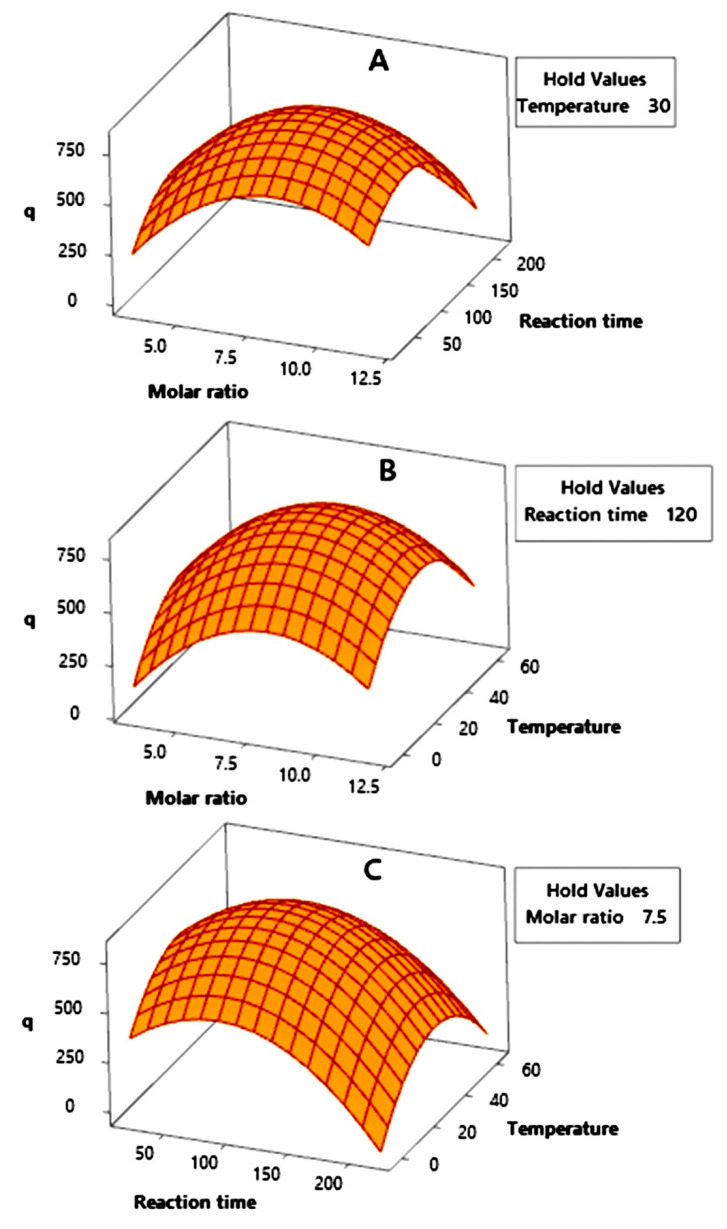
3D surface plots of the interaction effects between (**A**) the molar ratio and the reaction time, (**B**) the molar ratio and the temperature, and (**C**) the reaction time and the temperature on the synthesis of ZIF-L, as measured by the q response.

Figure 5 presents the 3D surface plots that describe the interaction effects between two out of three independent parameters whilst holding another parameter at its optimum level. As shown in Figure 5, the 3D surface plots already had their response peaks (q value). This means that the statistical design could represent the optimization study, as there was an escalation and depreciation of the response data.

Figure 5A describes the interaction between the molar ratio and the reaction time, with the temperature as the hold parameter. The highest point of the q value was obtained around a molar ratio of 8 and a reaction time of 100 min. This interpretation is continued in Figure 5B, which describes the interaction between the molar ratio and the temperature, with the reaction time as the hold parameter. This surface plot peak was obtained around a temperature of 30 °C and the same molar ratio as in Figure 5A. The interpretation of these 3D surface plots is confirmed by Figure 5C, in which the peak was obtained in the interaction between the same values of the reaction time and temperature. Furthermore, an optimization study was carried out to determine the real values of these three independent parameters in order to obtain the optimum q value.

#### 2.2.3. Optimization and Validation

**Figure 6:**
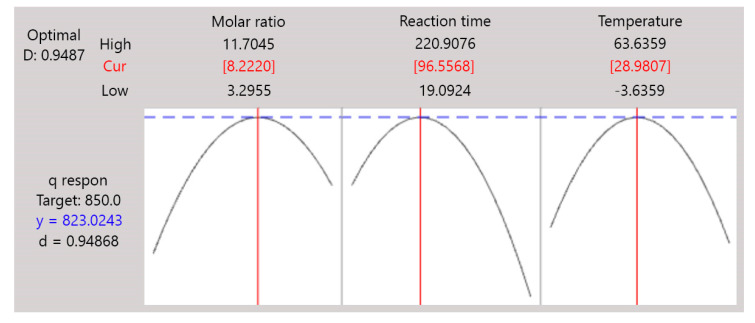
Optimization plots of the independent parameters for the synthesis of ZIF-L.

An optimization study was conducted to obtain the optimum q value based on the variations of three independent parameters. Here, the response’s target (or upper value) was set at 850.0 mg/g, or 85% of the initial CV concentration. The target set exceeded the removal efficiency of several dyes onto ZIFs in the previous study. The removal efficiency of rhodamine B (RB), methyl orange (MO) and methylene blue (MB) reached 50.0%, 50.5% and 46.6%, respectively [37]. As shown in Figure 6, the optimum ZIF-L could be obtained by a synthesis process using a molar ratio of 8.2220 for a reaction time of 97 min at a temperature of 29°C, where the highest q value of CV adsorption onto this ZIF-L reached 823.02 mg/g. This indicates that the removal efficiency of CV onto ZIF-L reached 82.3%, which was higher than the removal efficiency of RB, MO and MB using ZIFs from the previous study. As shown in Figure 6, the composite desirability of this optimization study reached 0.9487, close to 1, meaning that the result gave a satisfying optimization for this synthesis of ZIF-L. However, this optimization result still needed validation, and so the synthesis of ZIF-L was performed again using the optimal conditions. The validation was used to ensure the accuracy and consistency of the optimization result.

Table 3 represents the optimization study validation results, where the synthesis of ZIF-L was performed three times using a molar of 8.2220 for 97 min at 29 °C. The mean q value was 810.32 ± 9.25 mg/g from three runs of the synthesis process. The standard deviation of 9.25 mg/g (1.14%) was low enough to indicate this synthesis process’s consistency and repeatability. Furthermore, the optimization study error was 1.54 ± 1.12%, comparing the q values from the optimization and validation studies. The optimization study had a high accuracy, as proven by this low error value.

### 2.3. Characterizations of ZIF-L

#### 2.3.1. Scanning Electron Microscopy (SEM) Analysis

**Figure 7:**
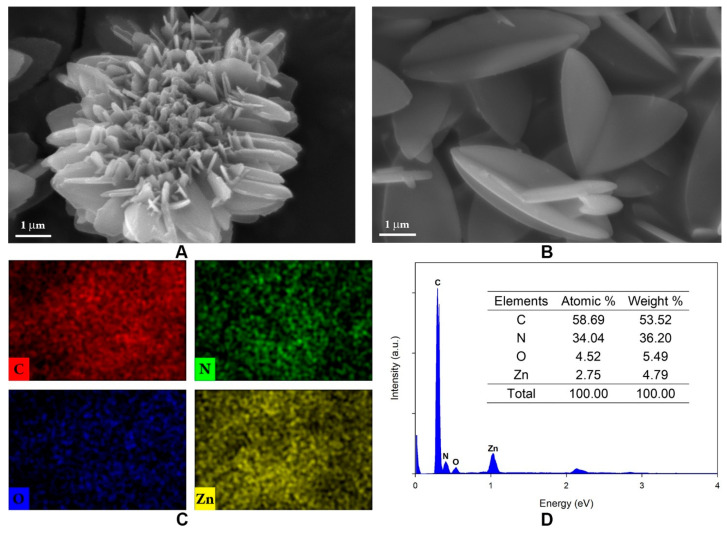
SEM photography of the ZIF-L synthesized (**A**) without the sonication step and (**B**) with the sonication step, along with (**C**) EDX mapping and (**D**) EDX analysis.

The characterizations were conducted for the ZIF-L obtained from the optimum synthesis process. Figure 7A,B shows the SEM images of the ZIF-L synthesized with and without the sonication step of the metal and ligand solutions. The synthesis with the sonication step produced a better shape of ZIF-L, the starfruit-like shape shown in Figure 7B. Figure 7A shows the aggregated ZIF-L, which would have a lower capability in further applications. In this study, the sonication step helped to produce the more homogeneous starfruit-like shape of ZIF-L. As seen in Figure 7B, the starfruit-like shape of ZIF-L was constructed by the 2D structures of ZIF-L which crossed each other. A previous study showed that MOFs with the same primary building units as ZIF-8, called ZIF-L, could be obtained at any range of molar ratio, including at a low molar ratio of ligand to metal [2,31,38]. However, the topotactic transition from the primary to the secondary building units is different, such that ZIF-L has a different crystal structure from ZIF-8 [2]. This synthesis of ZIF-L economically saved the amount of ligand used compared to the synthesis of ZIF-8, which commonly uses a molar ratio of ligand to metal of 70. However, ZIF-L retains excellent characteristics, even better than those of ZIF-8.

An EDX analysis was carried out to ensure the distribution of the main elements of ZIF-L. In the EDX mapping (Figure 7C), the red, green, blue and yellow colours indicated the distribution of the C, N, O and Zn elements, respectively. It showed a good distribution of the elements in the ZIF-L. Moreover, the quantitative amounts of each element are figured out in Figure 7D, where the mass percentages of C, N, O and Zn elements in the ZIF-L are shown in the inset table.

#### 2.3.2. X-ray Diffraction (XRD) Analysis

**Figure 8:**
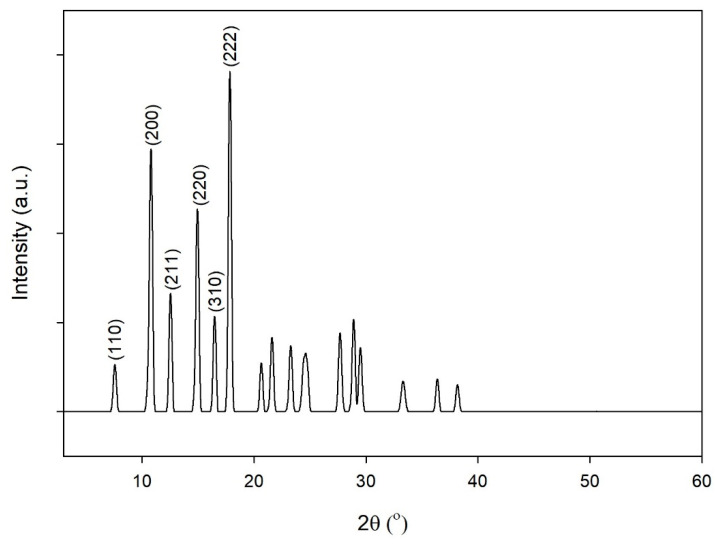
XRD spectra of ZIF-L.

The crystallinity of the ZIF-L was characterized using XRD analysis. As shown in Figure 8, the diffraction peaks of ZIF-L at the (110), (200), (211), (220), (310) and (222) planes were observed at 2θ values of 7.58°, 10.80°, 12.54°, 14.95°, 16.49° and 17.86°, respectively. This result had an excellent agreement with several reports from previous studies [14,20,28,34,39]. This proves that ZIF-L was successfully synthesized to have a high crystallinity.

#### 2.3.3. Fourier-Transform Infrared Spectroscopy (FTIR) Analysis

**Figure 9:**
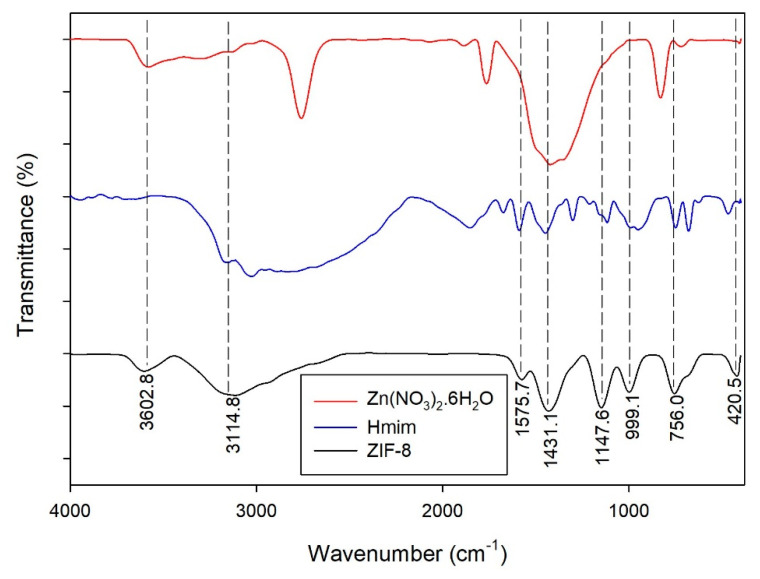
FTIR spectra of Zn(NO_3_)_2_.6H_2_O, Hmim and ZIF-L.

The functional groups of the ZIF-L were characterized using FTIR analysis. Figure 9 shows the FTIR spectra of the zinc precursor, ligand and ZIF-L. The spectrum of ZIF-L was similar to the spectra of the zinc precursor and ligand, meaning that the functional groups of ZIF-L were constructed from the functional groups of the zinc precursor and ligand. In the fingerprint region, several transmittance peaks were observed to identify the functional groups of the ZIF-L. The peaks observed at 420.5, 756.0 and 900–1350 cm^−1^ are associated with the Zn-N stretching of ZIF-L, the out-of-plane bending of the Hmim ring, and the in-plane bending of the Hmim ring, respectively [2,29,34,38,40]. 

In more detail, the in-plane bending of the Hmim ring consists of C-N vibration, C=N vibration, ring stretching, and N-H stretching assigned at 999.1, 1147.6, 1431.1 and 1575.1 cm^−1^, respectively [2,29,34,38,40]. Aside from the fingerprint region, two peaks were observed at 3114.8 and 3602.8 cm^−1^, which correspond to the aromatic C-H stretching of Hmim and the O-H stretching vibration of adsorbed water [2,29,40]. Here, the water as the solvent in the synthesis process is seen by the presence of an O-H stretching vibration peak in this spectrum of ZIF-L. There are no unidentified peaks in the spectrum of ZIF-L, and so no impurities affect the ZIF-L structure. A previous study reported the synthesis of ZIFs, especially ZIF-8, with the addition of a modulator, such as acetic acid [29]. Although the modulator could manage the formation of specific spherical ZIF-8, it gave additional functional groups in the ZIF-8 structure.

#### 2.3.4. Thermalgravimetric Analysis (TGA)

**Figure 10:**
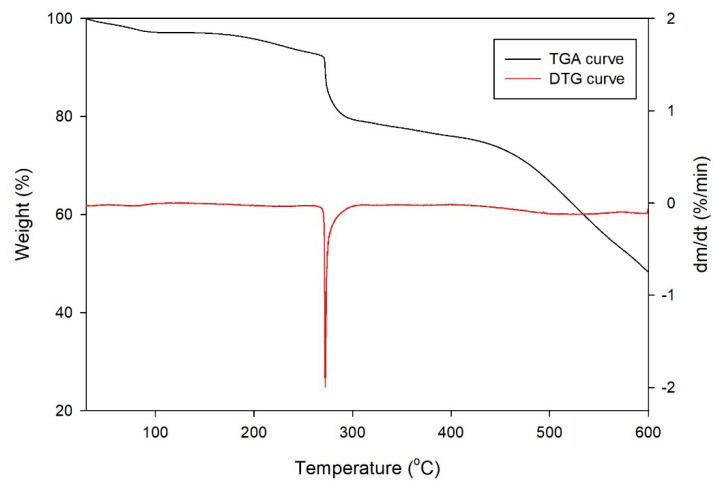
TGA and DTG curves of ZIF-L.

A TGA analysis was carried out for ZIF-L under a nitrogen flow, and the results are presented as the TGA and DTG curves in Figure 10. These results were compared with several previous studies, and they show a similar pattern of weight loss [34,38]. As shown in the TGA curve, the first weight loss gradually occurred at temperatures below 275 °C. This weight loss might be caused by the removal of moisture content and residual ligand from the ZIF-L surface. The removal of residual ligand was also indicated by the highest rate of weight loss at 272 °C, as shown by the DTG curve. Next, the good thermal stability of ZIF-L was observed at temperatures between 275 and 475 °C, where a long plateau was seen in the TGA curve. At temperatures above 475 °C, the ZIF-L structure began to collapse, as indicated by significant weight loss. However, this weight loss rate was minimal, as shown in the DTG curve, such that the collapse of the ZIF-L structure occurred gradually and took a long time in reasonably high temperatures.

## 3. Materials and Methods

### 3.1. Materials

The zinc nitrate hexahydrate (Zn(NO_3_)_2_.6H_2_O), 2-methylimidazole (Hmim) and crystal violet dye (CV) were purchased from Sigma-Aldrich (Singapore). All of the chemicals were analytical grade, and were used without further treatment.

### 3.2. Synthesis of ZIF-L

The ZIF-L was synthesized from Zn(NO_3_)_2_.6H_2_O as the metal source and Hmim as the ligand. A metal solution (0.075 M) was prepared in 40 mL distilled water. Another solution was prepared separately containing a specific ligand concentration in another 40 mL distilled water, in which the molar ratio of ligand to metal was varied by 5, 7.5 and 10. Both solutions were sonicated for 10 min, such that the compounds were be well ionized in the solvent. The metal solution was added dropwise with the ligand solution under stirring. The mixture was kept at various temperatures (10, 30, and 50 °C) for various reaction times (60, 120, and 180 min). In the meantime, the cloudy solution was obtained and then centrifuged for 10 min to separate the ZIF-L precipitation. The precipitation was washed with distilled water and ethanol (twice each), respectively, and dried overnight at 80 °C.

### 3.3. Adsorption of Crystal Violet Dye

Here, the amounts of adsorbed CV on the ZIF-L were used as the response in the statistical optimization of the ZIF-L preparation. A dye solution was prepared at a concentration of 1000 mg/L. The adsorption was carried out by adding 10 mg ZIF-L into 10 mL dye solution. It was shaken at room temperature for 24 h using a Memmert type WB-14 shaking water bath. After the adsorption, the solution was separated from the solid by centrifugation. A UV/Vis spectrophotometer was then used to measure the remaining CV concentration in the solution at a maximum wavelength of 590 nm. The amount of adsorbed CV on the ZIF-L was calculated by the following equation [41]:

(2) $$q=\left[\left(C_{i}-C_{r}\right)\times V/m\right]$$

where $C_{i}$ and $C_{r}$ are the initial and remaining CV concentrations (in mg/L), while V and m are the volume of the CV solution and the mass of ZIF-L. The adsorption was performed for all of the ZIF-L obtained from the combined synthesis parameters.

### 3.4. Design of the Experiment

#### 3.4.1. One-Factor-at-a-Time (OFAT) Method

The ZIF-L was prepared using the independent parameters mentioned in Table 4 with an OFAT method. Then, the adsorption of the CV was conducted in order to measure the q response for each ZIF-L. OFAT was used as the preliminary level determination before conducting the optimization analysis. Moreover, it was used as the early investigation of each parameter’s effect on the synthesis of ZIF-L.

#### 3.4.2. Response Surface Methodology

The central composite design (CCD) of the response surface methodology (RSM) using Minitab 19 statistical software was applied to design the synthesis of ZIF-L involving cube, axial and center points. There were three independent parameters used, i.e., the molar ratio of ligand to metal (r), reaction time (t) and temperature (T). Each parameter had three desired levels and two statistically additional levels, as described in Table 5. All of the combined parameters were repeated three times, such that there were 60 independent experiments in total. The RSM included the analysis of variance (ANOVA) in the determination of the significance of the parameters and their interactions.

#### 3.4.3. Optimization

RSM was employed in order to optimize the synthesis of ZIF-L statistically. Herein, the optimum condition was a synthesis condition that produced ZIF-L with the highest adsorption capacity towards crystal violet dye. The linear and quadratic effects of the independent parameters are mathematically expressed by the following equation [42,43]:

(3) $$Y=\alpha_{0}+\overset{i}{\underset{i=1}{\sum }}\alpha_{i}X_{i}+\overset{i}{\underset{i=1}{\sum }}\alpha_{ii}X_{i}^{2}+\overset{i}{\underset{i=1}{\sum }}\overset{j}{\underset{j=1}{\sum }}\alpha_{ij}X_{i}X_{j}$$

where Y is the predicted amount of dye adsorbed onto ZIF-L (in mg/L); $X_{i}$ and $X_{j}$ are the independent parameters used; and $\alpha_{0}$, $\alpha_{i}$, $\alpha_{ii}$, and $\alpha_{ij}$ are the intercept, linear, quadratic, and two-way interaction coefficients, respectively. Furthermore, this statistical analysis was carried out to represent the significance of the independent parameters (*p*-value < 0.05).

### 3.5. Characterizations

An X-ray diffraction (XRD) pattern was obtained by a PANalytical X’Pert Pro X-ray diffractometer with Cu Kα_1_ radiation (λ = 1.5406 Å). It was conducted at 40 kV and 30 mA using a step size of 0.02°/step. Scanning electron microscopy (SEM) was performed using a JSM-6390 field emission SEM, JEOL, Ltd., Japan, at an accelerating voltage of 10 kV and a working distance of 7.5 mm. The samples were prepared using a JFC-1200 coater, JEOL, Ltd., Japan, in an argon atmosphere. A Fourier transform infrared spectrophotometer (FTIR) was used to depict the spectra, indicating the samples’ functional groups. This analysis was carried out using an FTIR Shimadzu 8400S with the KBr pelleting method at a wavenumber range of 4000–400 cm^−1^. Thermalgravimetric analysis (TGA) was conducted using a Perkin Elmer Diamond TG/DTA thermal analyzer. It was carried out in a nitrogen gas flow of 150 mL/min at a constant heating and cooling rate in a temperature range of 27.5–600 °C.

## 4. Conclusions

The synthesis of ZIF-L was successfully carried out under the optimum conditions obtained from the optimization analysis. Statistically, the CCD in RSM was well fitted with the synthesis data, where the optimum condition was the synthesis process using a molar ratio of ligand to metal of 8.2220 for 97 min at 29 °C. This synthesis process is economically promising to produce high-quality ZIF-L, where the molar ratio used was lower than that in previous studies. The ZIF-L produced had a homogeneous starfruit-like shape with excellent crystallinity and thermal stability. It is a promising advanced material that can be used in various applications.

## Figures and Tables

**Table 1:** Design of the experiment on the ZIF-L synthesis, along with the experimental and predicted responses.

Run Order	Blocks	Points	Coded Parameters			Uncoded Parameter			q as the Response (mg/g)	
			r	t	T	r	t (min)	T (°C)	Experimental	Predicted
1	1	Cube	−1	−1	−1	5	60	10	522.32	522.36
2			+1	−1	−1	10	60	10	664.72	645.08
3			−1	+1	−1	5	180	10	336.47	337.96
4			+1	+1	−1	10	180	10	408.43	424.48
5			−1	−1	+1	5	60	50	498.08	479.12
6			+1	−1	+1	10	60	50	585.69	601.84
7			−1	+1	+1	5	180	50	350.26	353.92
8			+1	+1	+1	10	180	50	449.72	440.44
9		Axial	−1.68	0	0	3.30	120	30	437.14	439.11
10			+1.68	0	0	11.70	120	30	621.00	615.06
11			0	−1.68	0	7.5	19	30	599.45	624.11
12			0	+1.68	0	7.5	221	30	360.14	333.33
13			0	0	−1.68	7.5	120	−3.6	479.18	487.93
14			0	0	+1.68	7.5	120	63.6	477.81	464.99
15		Center	0	0	0	7.5	120	30	787.09	798.02
16			0	0	0	7.5	120	30	811.31	798.02
17			0	0	0	7.5	120	30	808.36	798.02
18			0	0	0	7.5	120	30	802.44	798.02
19			0	0	0	7.5	120	30	798.52	798.02
20			0	0	0	7.5	120	30	791.53	798.02
21	2	Cube	−1	−1	−1	5	60	10	547.40	522.36
22			+1	−1	−1	10	60	10	647.42	645.08
23			−1	+1	−1	5	180	10	350.84	337.96
24			+1	+1	–1	10	180	10	422.30	424.48
25			−1	−1	+1	5	60	50	489.60	479,12
26			+1	−1	+1	10	60	50	607.42	601.84
27			−1	+1	+1	5	180	50	356.08	353.92
28			+1	+1	+1	10	180	50	452.07	440.44
29		Axial	−1.68	0	0	3.30	120	30	438.61	439.11
30			+1.68	0	0	11.70	120	30	632.02	615.06
31			0	−1.68	0	7.5	19	30	627.63	624.11
32			0	+1.68	0	7.5	221	30	327.21	333.33
33			0	0	−1.68	7.5	120	– 3.6	490.08	487.93
34			0	0	+1.68	7.5	120	63.6	458.00	464.99
35		Center	0	0	0	7.5	120	30	795.24	798.02
36			0	0	0	7.5	120	30	798.38	798.02
37			0	0	0	7.5	120	30	805.92	798.02
38			0	0	0	7.5	120	30	795.41	798.02
39			0	0	0	7.5	120	30	816.45	798.02
40			0	0	0	7.5	120	30	793.29	798.02
41	3	Cube	−1	−1	−1	5	60	10	518.82	522.36
42			+1	−1	−1	10	60	10	616.05	645.08
43			−1	+1	−1	5	180	10	343.07	337.96
44			+1	+1	−1	10	180	10	423.69	424.48
45			−1	−1	+1	5	60	50	454.36	479,12
46			+1	−1	+1	10	60	50	605.29	601.84
47			−1	+1	+1	5	180	50	348.15	353.92
48			+1	+1	+1	10	180	50	407.54	440.44
49		Axial	−1.68	0	0	3.30	120	30	419.41	439.11
50			+1.68	0	0	11.70	120	30	617.88	615.06
51			0	−1.68	0	7.5	19	30	637.00	624.11
52			0	+1.68	0	7.5	221	30	324.36	333.33
53			0	0	−1.68	7.5	120	–3.6	486.33	487.93
54			0	0	+1.68	7.5	120	63.6	470.88	464.99
55		Center	0	0	0	7.5	120	30	780.22	798.02
56			0	0	0	7.5	120	30	780.15	798.02
57			0	0	0	7.5	120	30	788.73	798.02
58			0	0	0	7.5	120	30	794.52	798.02
59			0	0	0	7.5	120	30	810.04	798.02
60			0	0	0	7.5	120	30	806.20	798.02

**Table 2:** Analysis of variance results.

Source	Sum of Squares	Degree of Freedom	Mean Square	*F*-Value	*p*-Value
Model	1,688,160	11	153,469	894.54	0.000
Blocks	1271	2	635	3.70	0.032
r	112,111	1	112,111	653.47	0.000
t	306,223	1	306,223	1784.91	0.000
T	1907	1	1907	11.12	0.002
$r^{2}$	396,686	1	396,686	2312.20	0.000
$t^{2}$	551,006	1	551,006	3211.70	0.000
$T^{2}$	558,801	1	558,801	3257.14	0.000
r×t	1965	1	1965	11.45	0.001
r×T	94	1	94	0.55	0.463 *
t×T	5260	1	5260	30.66	0.000
Residual	8235	48	172		
Lack-of-fit	6585	33	200	1.81	0.110 *
Pure error	1650	15	110		
Total	1,696,395	59			

* not significant.

**Table 3:** Validation results of the optimization study on the synthesis of ZIF-L.

Runs	Molar Ratio of Ligand to Metal (r)	Reaction Time (t in min)	Temperature (T in °C)	q Response (mg/g)
1	8.2220	97	29	808.21
2				802.30
3				820.43
Mean value				810.32 ± 9.25
Optimized value				823.02
Error (%)				1.54 ± 1.12

**Table 4:** Parameters and levers of the OFAT experiments.

Parameters	Levels			
Molarratioofligandtometal (r)	2.5	5	7.5	10
Reactiontime (t in min)	30	60	120	180
Temperature (T, in °C)	10	30	50	70

**Table 5:** Uncoded and coded levels of the independent parameters.

Coded Levels	Parameters		
	Molar ratio of Ligand to Metal (r)	Reaction Time (t in min)	Temperature (T in °C)
−1.68	3.30	19	−3.6
−1	5	60	10
0	7.5	120	30
+1	10	180	50
+1.68	11.70	221	63.6

## Data Availability

Not applicable.

## Sample Availability

Samples of the compounds are available from the authors.

## References

[1] He Y., Shi L., Wang J., Yan J., Chen Y., Wang X., Song Y., Han G. (2020). UiO-66-NDC (1,4-naphthalenedicarboxilic acid) as a novel fluorescent probe for the selective detection of Fe^3+^. J. Solid State Chem..

[2] Jian M., Liu B., Liu R., Qu J., Wang H., Zhang X. (2015). Water-based synthesis of zeolitic imidazolate framework-8 with high morphology level at room temperature. RSC Adv..

[3] Sun S., Yang Z., Cao J., Wang Y., Xiong W. (2020). Copper-doped ZIF-8 with high adsorption performance for removal of tetracycline from aqueous solution. J. Solid State Chem..

[4] Zhao H., Li Q., Wang Z., Wu T., Zhang M. (2020). Synthesis of MIL-101(Cr) and its water adsorption performance. Microporous Mesoporous Mater..

[5] Zhuang S., Liu Y., Wang J. (2019). Mechanistic insight into the adsorption of diclofenac by MIL-100: Experiments and theoretical calculations. Environ. Pollut..

[6] Ediati R., Dewi S.K., Hasan M.R., Kahardina M., Murwani I.K., Nadjib M. (2019). Mesoporous HKUST-1 synthesized using solvothermal method. Rasayan J. Chem..

[7] Masoumi S., Tabrizi F.F., Sardarian A.R. (2020). Efficient tetracycline hydrochloride removal by encapsulated phosphotungstic acid (PTA) in MIL-53 (Fe): Optimizing the content of PTA and recycling study. J. Environ. Chem. Eng..

[8] Mao Y., Qi H., Ye G., Han L., Zhou W., Xu W., Sun Y. (2019). Green and time-saving synthesis of MIL-100(Cr) and its catalytic performance. Microporous Mesoporous Mater..

[9] Mon M., Bruno R., Tiburcio E., Casteran P.E., Ferrando-Soria J., Armentano D., Pardo E. (2018). Efficient Capture of Organic Dyes and Crystallographic Snapshots by a Highly Crystalline Amino-Acid-Derived Metal–Organic Framework. Chem. A Eur. J..

[10] Alivand M.S., Tehrani N.H.M.H., Shafiei-Alavijeh M., Rashidi A., Kooti M., Pourreza A., Fakhraie S. (2019). Synthesis of a modified HF-free MIL-101(Cr) nanoadsorbent with enhanced H_2_S/CH_4_, CO_2_/CH_4_, and CO_2_/N_2_ selectivity. J. Environ. Chem. Eng..

[11] Cui X., Sun X., Liu L., Huang Q., Yang H., Chen C. (2019). In-situ fabrication of cellulose foam HKUST-1 and surface modi fi cation with polysaccharides for enhanced selective adsorption of toluene and acidic dipeptides. Chem. Eng. J..

[12] Mon M., Bruno R., Ferrando-Soria J., Armentano D., Pardo E. (2018). Metal-organic framework technologies for water remediation: Towards a sustainable ecosystem. J. Mater. Chem. A.

[13] Chaturvedi G., Kaur A., Umar A., Khan M.A., Algarni H., Kansal S.K. (2020). Removal of fluoroquinolone drug, levofloxacin, from aqueous phase over iron based MOFs, MIL-100(Fe). J. Solid State Chem..

[14] He Y., Zeng L., Feng Z., Zhang Q., Zhao X., Ge S., Hu X., Lin H. (2020). Preparation, characterization, and photocatalytic activity of novel AgBr/ZIF-8 composites for water purification. Adv. Powder Technol..

[15] Chu F., Zheng Y., Wen B., Zhou L., Yan J., Chen Y. (2018). Adsorption of toluene with water on zeolitic imidazolate framework-8/graphene oxide hybrid nanocomposites in a humid atmosphere. RSC Adv..

[16] Elhussein E.A.A., Şahin S., Bayazit Ş.S. (2020). Removal of carbamazepine using UiO-66 and UiO-66/graphene nanoplatelet composite. J. Environ. Chem. Eng..

[17] Fan C., Dong H., Liang Y., Yang J., Tang G., Zhang W., Cao Y. (2019). Sustainable synthesis of HKUST-1 and its composite by biocompatible ionic liquid for enhancing visible-light photocatalytic performance. J. Clean. Prod..

[18] Pangestu T., Kurniawan Y., Soetaredjo F.E., Santoso S.P., Irawaty W., Yuliana M., Hartono S.B., Ismadji S. (2019). The synthesis of biodiesel using copper based metal-organic framework as a catalyst. J. Environ. Chem. Eng..

[19] Kumar A., Chowdhuri A.R., Kumari A., Sahu S.K. (2018). IRMOF-3: A fluorescent nanoscale metal organic frameworks for selective sensing of glucose and Fe (III) ions without any modification. Mater. Sci. Eng. C.

[20] Shi Z., Yu Y., Fu C., Wang L., Li X. (2017). Water-based synthesis of zeolitic imidazolate framework-8 for CO_2_ capture. RSC Adv..

[21] Tzitzios V., Kostoglou N., Giannouri M., Basina G., Tampaxis C., Charalambopoulou G., Steriotis T., Polychronopoulou K., Doumanidis C., Mitterer C. (2017). Solvothermal synthesis, nanostructural characterization and gas cryo-adsorption studies in a metal–organic framework (IRMOF-1) material. Int. J. Hydrog. Energy.

[22] Simon M.A., Anggraeni E., Soetaredjo F.E., Santoso S.P., Irawaty W., Thanh T.C., Hartono S.B., Yuliana M., Ismadji S. (2019). Hydrothermal Synthesize of HF-Free MIL-100(Fe) for Isoniazid-Drug Delivery. Sci. Rep..

[23] Strzempek W., Menaszek E., Gil B. (2019). Fe-MIL-100 as drug delivery system for asthma and chronic obstructive pulmonary disease treatment and diagnosis. Microporous Mesoporous Mater..

[24] Safaei M., Foroughi M.M., Ebrahimpoor N., Jahani S., Omidi A., Khatami M. (2019). A review on metal-organic frameworks: Synthesis and applications. Trends Anal. Chem..

[25] Son Y.R., Ryu S.G., Kim H.S. (2020). Rapid adsorption and removal of sulfur mustard with zeolitic imidazolate frameworks ZIF-8 and ZIF-67. Microporous Mesoporous Mater..

[26] Abdelhamid H.N., Zou X. (2018). Template-free and room temperature synthesis of hierarchical porous zeolitic imidazolate framework nanoparticles and their dye and CO_2_ sorption. Green Chem..

[27] Li R., Li W., Jin C., He Q., Wang Y. (2020). Fabrication of ZIF-8@TiO_2_ micron composite via hydrothermal method with enhanced absorption and photocatalytic activities in tetracycline degradation. J. Alloys Compd..

[28] Lee Y., Jang M., Cho H., Kwon H., Kim S., Ahn W. (2015). ZIF-8: A comparison of synthesis methods. Chem. Eng. J..

[29] Santoso E., Ediati R., Istiqomah Z., Sulistiono D.O., Nugraha R.E., Kusumawati Y., Bahruji H., Prasetyoko D. (2021). Facile synthesis of ZIF-8 nanoparticles using polar acetic acid solvent for enhanced adsorption of methylene blue. Microporous Mesoporous Mater..

[30] Huang C., Zhang H., Zheng K., Zhang Z., Jiang Q., Li J. (2021). Two-dimensional hydrophilic ZIF-L as a highly-selective adsorbent for rapid phosphate removal from wastewater. Sci. Total Environ..

[31] Zhang F., Dou J., Zhang H. (2018). Mixed membranes comprising carboxymethyl cellulose (as capping agent and gas barrier matrix) and nanoporous ZIF-L nanosheets for gas separation applications. Polymers.

[32] Zhong Z., Yao J., Chen R., Low Z., He M., Liu J.Z., Wang H. (2015). Oriented two-dimensional zeolitic imidazolate framework-L membranes and their gas permeation properties. J. Mater. Chem. A.

[33] Chen R., Yao J., Gu Q., Smeets S., Baerlocher C., Gu H., Zhu D., Morris W., Yaghi O.M., Wang H. (2013). A two-dimensional zeolitic imidazolate framework with a cushion-shaped cavity for CO_2_ adsorption. Chem. Commun..

[34] Khan I.U., Othman M.H.D., Ismail A.F., Ismail N., Jaafar J., Hashim H., Rahman M.A., Jilani A. (2018). Structural transition from two-dimensional ZIF-L to three-dimensional ZIF-8 nanoparticles in aqueous room temperature synthesis with improved CO_2_ adsorption. Mater. Charact..

[35] Gross A.F., Sherman E., Vajo J.J. (2012). Aqueous room temperature synthesis of cobalt and zinc sodalite zeolitic imidizolate frameworks. Dalt. Trans..

[36] Kida K., Okita M., Fujita K., Tanaka S., Miyake Y. (2013). Formation of high crystalline ZIF-8 in an aqueous solution. CrystEngComm.

[37] Tran B.L., Chin H.Y., Chang B.K., Chiang A.S.T. (2019). Dye adsorption in ZIF-8: The importance of external surface area. Microporous Mesoporous Mater..

[38] Ding B., Wang X., Xu Y., Feng S., Ding Y., Pan Y., Xu W., Wang H. (2018). Hydrothermal preparation of hierarchical ZIF-L nanostructures for enhanced CO_2_ capture. J. Colloid Interface Sci..

[39] Valencia L., Abdelhamid H.N. (2019). Nanocellulose leaf-like zeolitic imidazolate framework (ZIF-L) foams for selective capture of carbon dioxide. Carbohydr. Polym..

[40] Mahmoodi N.M., Keshavarzi S., Oveisi M., Rahimi S., Hayati B. (2019). Metal-organic framework (ZIF-8)/inorganic nanofiber (Fe_2_O_3_) nanocomposite: Green synthesis and photocatalytic degradation using LED irradiation. J. Mol. Liq..

[41] Wijaya C.J., Ismadji S., Aparamarta H.W., Gunawan S. (2020). Hydrophobic modification of cellulose nanocrystals from bamboo shoots using rarasaponins. ACS Omega.

[42] Wijaya C.J., Ismadji S., Aparamarta H.W., Gunawan S. (2019). Optimization of cellulose nanocrystals from bamboo shoots using Response Surface Methodology. Heliyon.

[43] Yuliana M., Sutrisno R.J., Hermanto S., Ismadji S., Wijaya C.J., Santoso S.P., Soetaredjo F.E., Ju Y.-H. (2020). Hydrophobic Cetyltrimethylammonium Bromide-Pillared Bentonite as an Effective Palm Oil Bleaching Agent. ACS Omega.

